# The Fracture Load as a Function of the Material Thickness: The Key to Computing the Strength of Monolithic All-Ceramic Materials?

**DOI:** 10.3390/ma16051997

**Published:** 2023-02-28

**Authors:** Josef Schweiger, Kurt-Jürgen Erdelt, Tobias Graf, Thomas Sciuk, Daniel Edelhoff, Jan-Frederik Güth

**Affiliations:** 1 Department of Prosthetic Dentistry, University Hospital, LMU Munich, 80336 Munich, Germany; 2 Department of Prosthodontics, Center for Dentistry and Oral Medicine (Carolinum), Goethe University Frankfurt, 60596 Frankfurt am Main, Germany; 3 Thomas Sciuk, Private Practice Dr Thomas Sciuk, Prinzregentenstrasse 8, 86150 Augsburg, Germany

**Keywords:** all-ceramics, digital workflow fracture load material thickness, monolithic restorations, regression analysis

## Abstract

The thickness of a material has a significant impact on its fracture load. The aim of the study was to find and describe a mathematical relationship between the material thickness and the fracture load for dental all-ceramics. In total, 180 specimens were prepared from a leucite silicate ceramic (ESS), a lithium disilicate ceramic (EMX), and a 3Y-TZP zirconia ceramic (LP) in five thicknesses (0.4, 0.7, 1.0, 1.3, and 1.6 mm; n = 12). The fracture load of all specimens was determined using the biaxial bending test according to the DIN EN ISO 6872. The regression analyses for the linear, quadratic, and cubic curve characteristics of the materials were conducted, and the cubic regression curves showed the best correlation (coefficients of determination (R^2^): ESS R^2^ = 0.974, EMX R^2^ = 0.947, LP R^2^ = 0.969) for the fracture load values as a function of the material thickness. A cubic relationship could be described for the materials investigated. Applying the cubic function and material-specific fracture-load coefficients, the respective fracture load values can be calculated for the individual material thicknesses. These results help to improve and objectify the estimation of the fracture loads of restorations, to enable a more patient- and indication-centered situation-dependent material choice.

## 1. Introduction

Patients increasingly expect high-quality dental restorations in terms of function, tolerability, longevity, and esthetics [1,2,3]. The continuously growing number of all-ceramic restorative materials makes it more challenging for dentists and dental technicians to select the most suitable material for indirect prosthetic restorations [3,4,5]. The restorative materials should imitate natural teeth as closely as possible to meet our patients’ expectations in terms of stability, function, and esthetics [6,7], and the material choice influences the long-term success of a restoration. Aside from the biomechanical properties, the optical properties, and in particular, the translucency, are of decisive importance to mimic the appearance of natural teeth, and to achieve an esthetically satisfactory outcome [8,9].

Over the course of the steadily evolving CAD-CAM technology, a trend towards monolithic restorations in all material classes can be observed. Besides the digital feasibility, economic aspects, as well as an extension of the clinical indications and applications—especially for full ceramic restorations—are the obvious reasons for this development.

However, to fulfill the abovementioned criteria and demands, the biomechanical and optical properties of the restorative materials for monolithic use need to be well balanced against each other [3].

Various studies have pointed out that the thickness of the restorative materials has a determining influence on the translucency of the dental restorations, significantly affecting the esthetic outcomes [9,10,11,12]. In other words, the lower the thickness of the restorative material, the higher the translucency, but the lower the mechanical strength [13,14,15,16,17]. Material thickness plays a key role in ensuring that dentures remain functional over an extended period and without complications. At the same time, a minimally invasive preparation should be performed to minimize the biological costs and complications [18,19]. Practitioners are expected to provide restorations with pleasing esthetics and, at the same time, sufficient strength, while ensuring minimally invasive preparations—contradictory requirements in daily practice. Since changes in material thickness could have a major effect on the strength of the restorative material, the relationship between the material thickness and the long-term stability of the restorative materials is of enormous clinical importance.

Previous studies have confirmed that an increase in material thickness correlates to higher fracture load. However, thus far, no mathematical relationship between the material thickness and fracture load has been specifically reported for dental ceramics [13,14,15,16,17]; some examinations already exist, which describe a cubic relationship of the fracture load on the material thickness for resin-based CAD-CAM restorative materials [20]. Against this background, it seems to be of great importance to investigate whether comparable correlations occur for ceramic materials, which are far more frequently used for prosthodontic restorations [21].

Such a mathematical relationship can provide additional information when fabricating dental restorations, combining factors such as the preparation depths and thus the material layer thicknesses with the fracture load of the preferred restoration material. Since the chemical structures and, hence, the strength parameters of dental ceramics vary significantly [2,22], the present study was undertaken to determine whether specific fracture load curves as a function of material thickness could be determined for three groups of all-ceramics (leucite-reinforced silicate ceramics, lithium disilicate ceramics, and oxide ceramics).

The aim of the present study was to find a mathematical relationship between the material thickness and the fracture load and to develop a generally applicable equation with associated material-specific parameters (fracture-load coefficients) for various all-ceramics.

## 2. Materials and Methods

The present study examined a leucite-reinforced silicate ceramic (IPS Empress CAD (“ESS”), Ivoclar Vivadent, Schaan, Liechtenstein), a lithium disilicate ceramic (IPS e.max CAD (“EMX”), Ivoclar Vivadent, Schaan), and a 3Y-TZP oxide ceramic (Lava Plus (“LP”), 3M, Seefeld, Germany) (Table 1).

**Figure 1:**
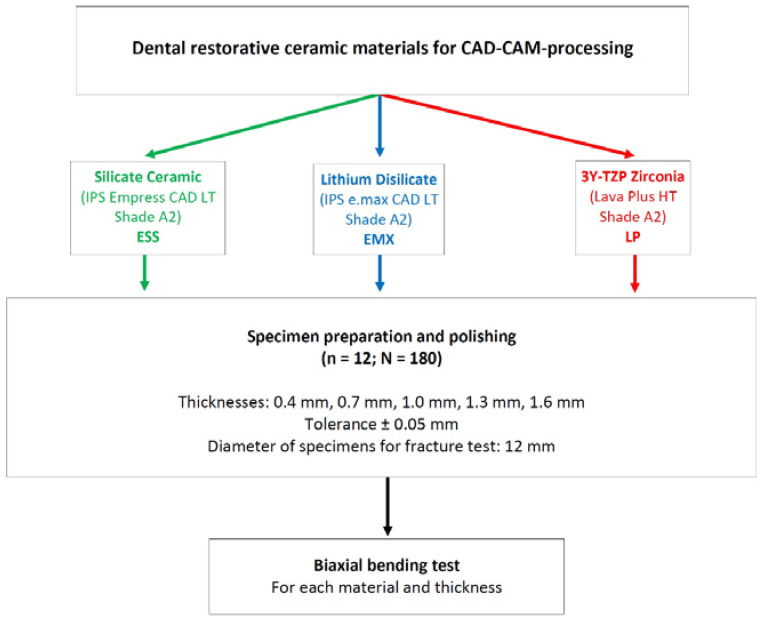
Study design and setup.

For each material, 60 specimens were produced and, in turn, subdivided into 5 different material thicknesses of 12 specimens each. The specimens were 0.4, 0.7, 1.0, 1.3, or 1.6 mm thick (Figure 1).

### 2.1. Specimen Production

The ESS and EMX were available in the form of shaded CEREC blocks; the LP was available as a circular blank (diameter 98 mm). Cylinders with a diameter of either 12 mm (ESS, EMX) or 15 mm (LP) were milled using the milling machines Cerec inLab MC XL (Dentsply Sirona, York, PA; ESS, EMX) or Ceramill Motion 2 (Amann Girrbach, Pforzheim, Germany; LP). The cylinders were cut under irrigation to 0.6 mm, 0.9 mm, 1.2 mm, 1.5 mm, and 1.8 mm, respectively, using a precision cutting machine (Secotom 50; Struers, Willich, Germany).

The EMX and LP required thermal pretreatment before trimming and polishing. The LP was stained with the manufacturer-specific liquid (FS 3 Lava Frame Shading Liquid; 3M) and sintered in the sintering furnace (Sintramat; Ivoclar Vivadent) at 1450 °C for two hours according to the manufacturer’s specifications. The EMX was crystallized at 840 °C (Programat EP 5000 furnace, Ivoclar Vivadent), also according to the manufacturer’s specifications.

**Figure 2:**
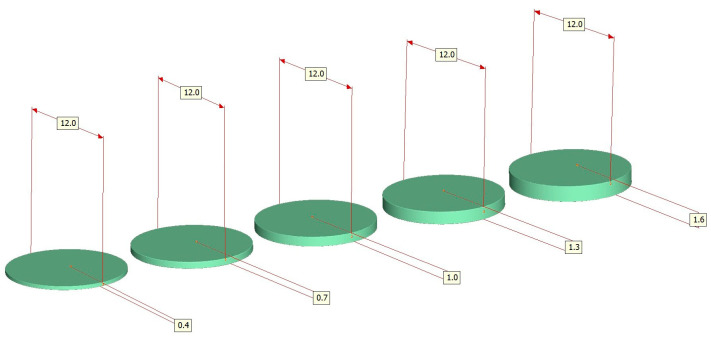
The shape and dimension of the test specimens used.

The specimens were adjusted to the final thicknesses by grinding in an automatic polishing machine (Abramin; Struers) under controlled conditions with water cooling (MD Rondo; Struers; 40 and 20 µm grid) and subsequently polished with a polishing wheel (MD Largo; Struers) in combination with diamond suspensions (DP-Suspension M; Struers) of different grit sizes (9, 3, and 1 µm). Grinding and polishing were performed on both planar sides of the specimens, resulting in final thicknesses of 0.4, 0.7, 1.0, 1.3, and 1.6 mm, after finishing and polishing with a maximum admissible dimensional error of ±0.05 mm (Figure 1 and Figure 2). The thickness measurements were assessed using a micrometer screw (Mitotoyu IP65, Tokyo, Japan; accuracy: ±1µm) at the center of the specimens and at their edges in the sectors 0°, 90°, 180°, and 270°. After polishing, all specimens were cleaned in an ultrasonic cleaning unit (T-14; L & R, Kearney, NJ, USA).

### 2.2. Fracture Load Test

The fracture load was determined using the biaxial bending test (DIN EN ISO 6872) [23]. The surfaces and diameters of the specimens were determined by the requirements of the DIN standard, so that the values of the 1.0 mm thick specimens could be used to determine the flexural strength.

**Figure 3:**
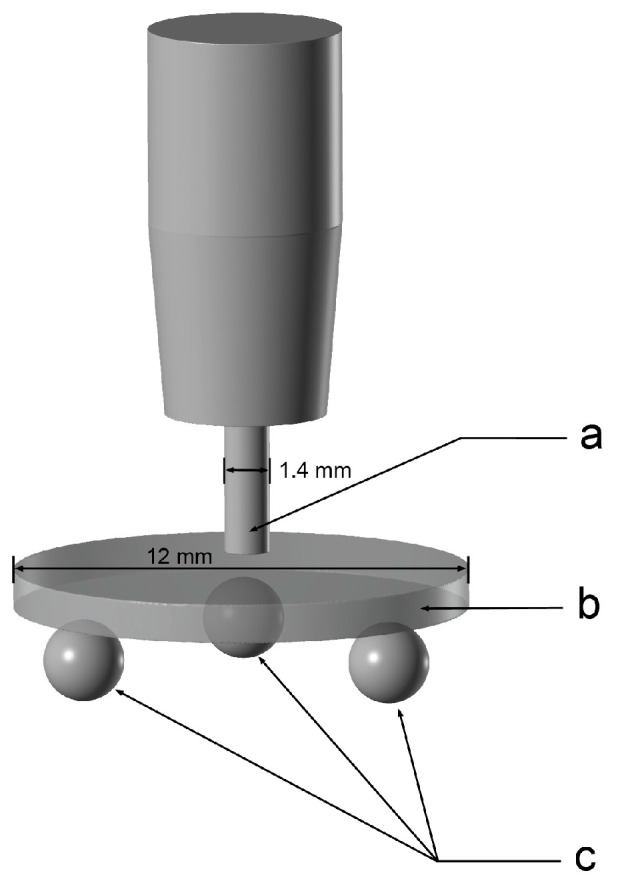
Schematic drawing of the biaxial bending test (DIN EN ISO 6872) (a. steel piston; b. test specimen; c. supporting spheres placed in a circular manner at 120° from each other).

The fracture load tests were carried out in the universal testing machine (UPM 1445; Zwick, Ulm, Germany), with a loading speed of 0.5 mm/min. The specimens were positioned on three supporting balls with a diameter of 3.2 ± 0.5 mm, which were angled at 120° from each other on a support disc with a diameter of 10.0 mm. The specimen was concentrically placed on these bearings and loaded at its center with a steel piston (diameter 1.4 mm) (Figure 3).

### 2.3. Data Analysis

The fracture load values were imported into a statistics program (Statistics 25.0; SPSS, Stanford, CA). The Kolmogorov–Smirnov test was applied to test for a normal distribution. The fracture loads of individual materials and the standard deviations were calculated by descriptive statistics. One-way ANOVA followed by Scheffé’s post hoc test determined the relationship between the thickness and the fracture load. Regression analysis was used to determine the fit (coefficients of determination (R^2^)) for the linear, quadratic, and cubic curve shapes versus the material thickness for each material group. To calculate the regression curves of the three different materials, the fracture load at a material thickness of 0.0 mm was defined as an additional supporting point. This fracture load value was 0 N for all materials by definition.

## 3. Results

The Kolmogorov–Smirnov revealed a normal distribution for all groups. Table 2 shows the mean values and standard deviations of the fracture load tests as well the flexural strengths calculated according to the DIN EN ISO 6872 [23] for all tested materials.

**Figure 4:**
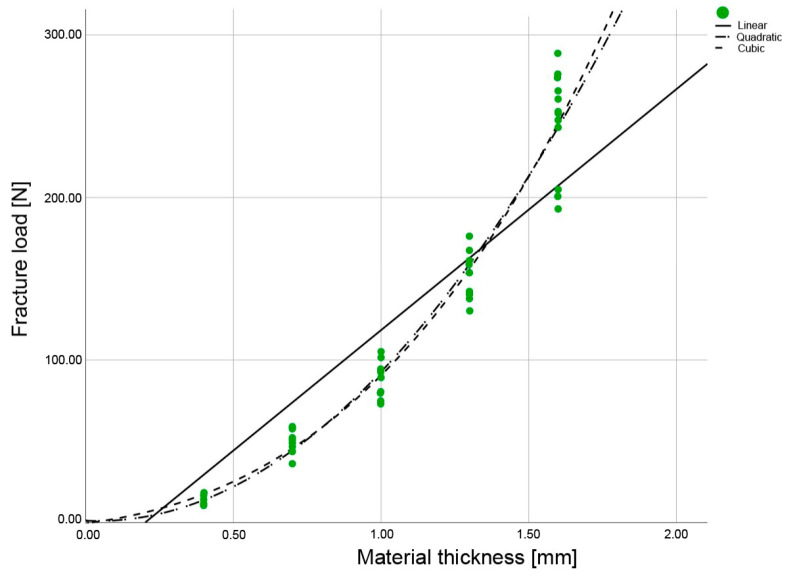
Regression curves of the fracture load values for the IPS Empress CAD LT A2 (ESS), with specimens in the five different material thicknesses examined.

**Figure 5:**
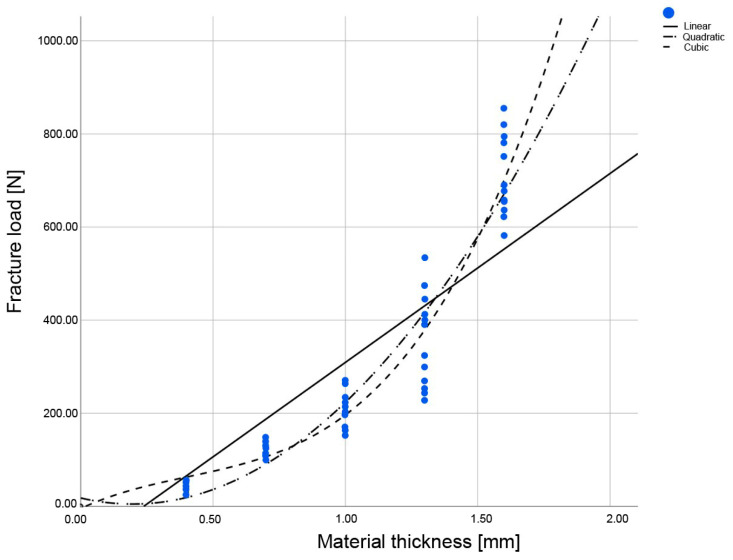
Regression curves of the fracture load values for the IPS e.max CAD LT A2 (EMX), with specimens in the five different material thicknesses examined.

**Figure 6:**
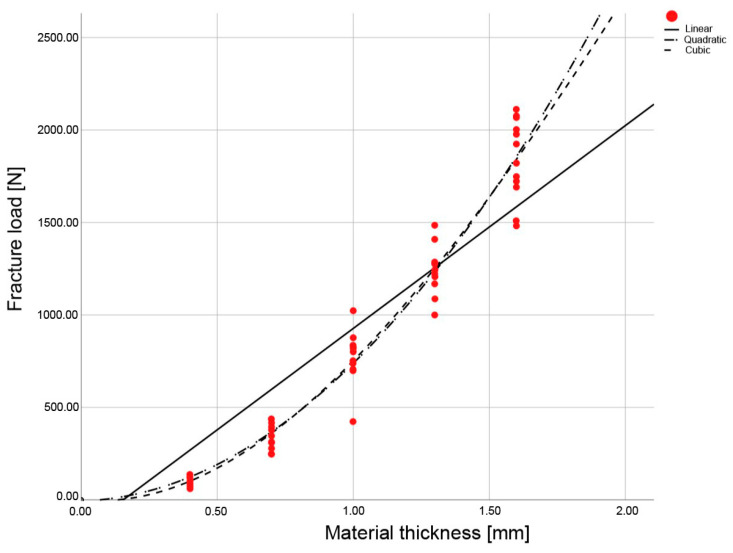
Regression curves of the fracture load values for the Lava Plus HT A2 (LU), with specimens in the five different material thicknesses examined.

**Figure 7:**
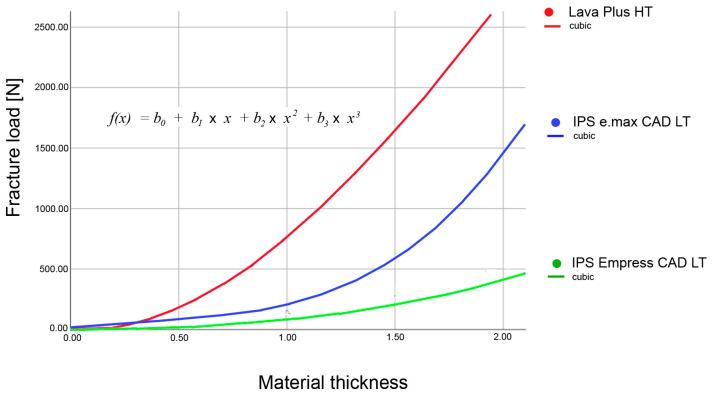
Cubic regression curves for all three materials examined.

For the LP, the fit was identical for the quadratic and cubic regression curves; for the ESS and EMX, the cubic curve exhibited the best alignment (R^2^) (Figure 4, Figure 5 and Figure 6, Table 3). Thus, the cubic regression curves most accurately described the fracture load values within the thicknesses from 0 mm to 1.6 mm, for the materials investigated (Figure 7). Using the resulting material-specific fracture-load coefficients b_0_, b_1_, b_2_, and b_3_ (Table 4) and the cubic fracture-load equation, the fracture load of the materials can be calculated for any individual material thickness.

Fracture-load Equation (1)

f (x) = b_0_ + b_1_x+ b_2_x^2^ + b_3_x^3^, (1)

where

f(x) is the fracture strength;

x is the material thickness;

b_0_, b_1_, b_2_, and b_3_ are the fracture load coefficients.

The linear (2) and quadratic (3) formulas can be defined as:

f(x) = b_0_ + b_1_x, (2)

f(x) = b_0_ + b_1_x + b_2_x^2^, (3)

where

f(x) is the fracture strength;

x is the material thickness;

b_0_, b_1_, and b_2_ are the fracture load coefficients.

The one-way ANOVA followed by Scheffé’s post hoc test showed differences between the materials, i.e., significantly different curve shapes for the three tested materials.

## 4. Discussion

The present study found that the cubic regression curves described the dependency of the fracture loads on the thickness for different all-ceramic materials. These findings must not be interpreted as the fracture loads for specific restorations. Rather, it could be the basis for future automated calculations to better estimate the individual strength of full ceramic restorations due to their individual design and geometry. However, some critical remarks need to be mentioned and discussed.

The present study, as many others, applied the biaxial bending test of the DIN EN ISO 6872 [20,24,25,26]. First of all, it is important to note that the flexural strength is determined using a standard with defined specimens and given in megapascals [MPa] [27]. Fracture loads, on the other hand, are based on the individual geometries of specimens or restorations. What was measured was the specimen’s fracture load in Newtons [N]. Therefore, the fracture loads for different specimen geometries are comparable only to a limited extent and are best considered for guidance purposes only [27]. For the classification and estimation of the magnitude, the mean masticatory forces of 298.8 N are given in the literature by Körber and Ludwig [28]. Nevertheless, the amount of maximal masticatory forces in the oral cavity depends on the localization and the individuum and can reach up to 738 N in the first molar region [29,30].

To establish a mathematical relationship, the method of best fit (R^2^) can be used to assess the shape of the regression curve. The closer the R² is to 1, the better the regression curve fits the determined measuring points.

To improve the significance of the R^2^ fit and, hence, the curve itself, the number of supporting points could be further increased. In the present study, this was implemented with six support points (0.0, 0.4, 0.7, 1.0, 1.3, and 1.6 mm). A standardized manual control mechanism was applied to ensure that the standard deviations within the measurement series were kept to a minimum, which probably had a positive impact on the fit of the curves. The smaller the standard deviations in the fracture load tests, the fewer support points are theoretically needed to determine the material-specific cubic coefficients. In theory, the zero point and three other thickness values would suffice to determine the material-specific fracture load coefficients. The method of least squares was used to determine the regression curves, minimizing the deviation error between the measurement points and the regression curve. The regression curve obtained was the one that had the smallest deviation error considering all the measured points.

All the materials were mechanically polished to a high gloss as per DIN EN ISO 6872. This seems relevant, as Wiedenmann et al. demonstrated for lithium disilicate and zirconia ceramics (and others) that the surface pretreatment after grinding is of crucial importance to ensure the optimum mechanical properties for a ceramic material [31]. Alakad et al. found that the combination of polishing and glazing surface treatment resulted in a significantly higher flexural strength than polishing alone. This study group also found a mean value of 268 MPa for the polished EMX specimens, which was within the range of the present study and thus below the manufacturer’s specification [32].

The fracture load curves (Figure 7) of the individual materials showed that the curve characteristics of the materials depended on their flexural strengths (Table 3). The present study resulted in flatter curve characteristics for materials with lower flexural strengths. It can be concluded that for all-ceramics with low flexural strengths, an increase in the material thickness will result in a smaller increase in the fracture load than for materials with high flexural strengths. This means that in clinical situations where the available vertical dimension is limited due to tooth preparation or other factors but where a higher fracture load is still required, materials with a higher flexural strength (e.g., EMX and LP) should be used. This is the only way to achieve a significant increase in the fracture load, whereas “moderate re-preparation” does not appear to be useful or effective when space is limited.

Misconceptions of the resulting fracture loads might be one of the reasons why ceramic fractures have been reported for monolithic restorations. Lithium disilicate and zirconia ceramics in their monolithic forms generally show low fracture rates [33,34]. Sulaiman et al. reported from 36,198 monolithic lithium disilicate single crowns a fracture rate of 0.96% within an observation period of up to 7.5 years in their retrospective study. From 77,411 monolithic zirconia single crowns, a percentage of 0.54% fractured, whereas the layered zirconia restorations showed 2.83%, a significantly higher fracture/ chipping rate [34].

It is conceivable that one might assume a linear relationship between the thickness and fracture load of a restoration and that, consequently, “reducing” the restoration´s thickness would halve its fracture load. However, this is an assumption that the present investigation has firmly refuted. This also means that estimating the cubic correlations is more complex during the daily routine. In the previous study of Graf et al., these cubic correlations were also found for other tooth-colored restoration materials, namely resin matrix ceramics (RMCs) and PMMA [20]. The current results are based on highly standardized specimens to find the fundamental constants to better understand the materials’ behavior that might be the basis for future individual geometry- and design-based calculations of the fracture strength. So, the mathematical relations described here offer a basis to further develop computed models that help resolve the ensuing problem.

Table 5 shows the fracture load values for the tested ceramics at a given layer thickness, allowing more precise estimations as to which material would be appropriate to use in a specific situation. In our digital age, however, it makes sense to combine the fracture-load equation with CAD-CAM technology. This means that the cubic function found—including the material-specific fracture-strength coefficients—should be implemented in the CAD design software; the fracture load values should be calculated automatically during the design process. This allows more predictable restorations to be designed considering the individual risk of premature failure and to possibly warn the operator. Overall, this might promote the standardization of work processes and optimize the quality management aspects in the manufacturing chain [35]. Since material-specific fracture-strength coefficients are already available for RMCs as well [20], it would seem a good idea to also determine these coefficients for other materials so that a universal application might be possible in future. The required experimental setup is straightforward, since the material effort is low, the geometry of the test specimens can easily be produced in a CAD-CAM based workflow, and the polishing steps can be conducted using tools readily available in dental engineering.

**Figure 8:**
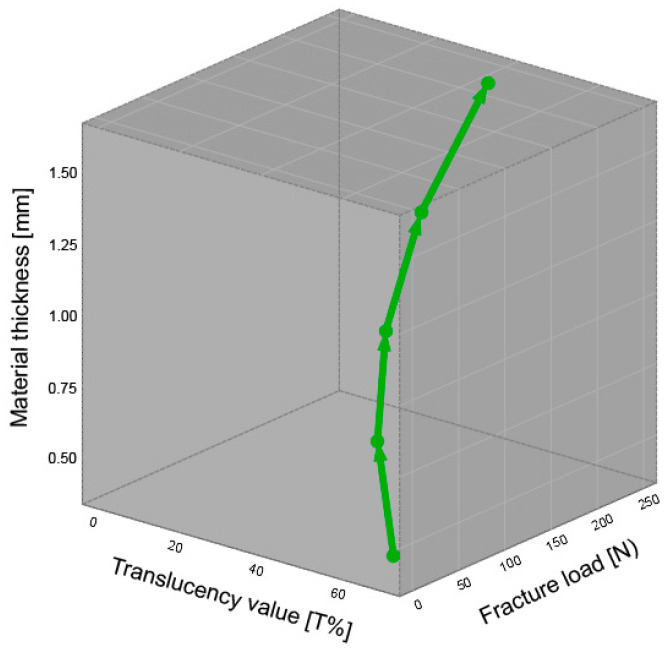
Three-dimensional regression curve of IPS Empress CAD LT A2 for the translucency, material thickness, and fracture load.

Previously, it was shown that the transmission of light in dental ceramics depends logarithmically on the material thickness [9]. Combining the findings obtained in the present study with the results from an evaluation of translucency leads to a three-dimensional regression curve, representing a relationship between the fracture load and the translucency of a given material dependent on its thickness (Figure 8). This could provide additional insights with regard to material selection and help optimize the choice of materials for practical application.

## 5. Conclusions

Within the limitations of this study, a mathematical correlation between the material thickness and the fracture load was described for the all-ceramic materials investigated. Applying a cubic function and material-specific fracture-load coefficients, the respective fracture load values were calculated for the individual material thicknesses. These results are a basis to further improve and objectify the estimation of the fracture loads of restorations and to enable a more patient- and indication-centered situation-dependent material choice. Against this background, an implementation in CAD systems and an extension of study to cover additional materials seems more than reasonable.

## Figures and Tables

**Table 1:** Details of the dental ceramics evaluated.

Material	Code	Material Class	Manufacturing Process	Batch #	Manufacturer
IPS Empress CAD LT A2	ESS	Leucite reinforced silicate ceramic	CAD-CAM	U36021	Ivoclar, Schaan, Liechtenstein
IPS e.max CAD LT A2	EMX	Lithium Disilicate ceramic	CAD-CAM	U32750	Ivoclar, Schaan, Liechtenstein
LAVA Plus HT A2	LP	3Y-TCP Zirconia	CAD-CAM	601514	3M, St. Paul, MN

**Table 2:** Descriptive statistics and flexural strengths [MPa] of the tested materials, determined according to the DIN EN ISO 6872; the same letters A to O indicate no statistically significant differences of the fracture load tested by Scheffé’s post hoc test.

Material	Material Thickness					Flexural Strength ^1^ (Mean ± SD) [MPa]
	0.4 mm	0.7 mm	1.0 mm	1.3 mm	1.6 mm	
	Fracture Load (Mean ± SD) [N]					
ESS	14.65 ± 2.25 (A)	48.65 ± 6.17 (B)	90.92 ± 11.32 (C)	154.02 ± 13.50 (D)	246.45 ± 31.35 (E)	136.25 ± 16.60
EMX	41.29 ± 7.03 (F)	124.95 ± 15.60 (G)	209.29 ± 36.39 (H)	355.65 ± 100.76 (I)	709.00 ± 87.37 (J)	288.41 ± 54.93
LP	104.97 ± 19.71 (K)	348.76 ± 68.40 (L)	768.17 ± 140.31 (M)	1244.02 ± 129.01 (N)	1844.81 ± 216.77 (O)	1204.75 ± 213.90

^1^ values of 1.0 mm thickness were used to determine the flexural strength.

**Table 3:** R^2^ values of the linear, quadratic, and cubic regression curves.

Material	Linear	Quadratic	Cubic
ESS	0.882	0.973	0.974 ^1^
EMX	0.802	0.931	0.947 ^1^
LP	0.895	0.969 ^1^	0.969 ^1^

^1^ fracture load values that fit the curves best.

**Table 4:** The material-specific fracture load coefficients b_0_, b_1_, b_2_, and b_3_ for the cubic, quadratic, and linear equations. (*p* ≤ 0.05).

Material	Material-Specific Cubic Fracture Load Coefficients [Means ± SD]				Sig. ^1^
	b_0_ (N)	b_1_ (N/mm)	b_2_ (N/mm^2^)	b_3_ (N/mm^3^)	
**ESS cubic**	**−0.50 ± 3.72**	**23.29 ± 23.75**	**44.75 ± 38.07**	**23.03 ± 15.80**	**0.000**
ESS quadratic	1.08	−7.80	99.49		0.000
ESS linear	−29.83	148.23			0.000
**EMX cubic**	**−4.87 ± 15.97**	**263.94 ± 97.32**	**−352.46 ± 153.94**	**289.65 ± 63.60**	**0.000**
EMX quadratic	17.86	−132.91	338.43		0.000
EMX linear	−96.80	405.94			0.000
**LP cubic**	**0.25 ± 34.30**	**−136.64 ± 19.98**	**1028.33 ± 131.87**	**−139.57 ± 34.30**	**0.000**
LP quadratic	−11.50	56.30	694.62		0.000
LP linear	−257.96	1171.67			0.000

^1^ significance of correlation.

**Table 5:** The calculated fracture loads for the three materials investigated.

Material	The Calculated Fracture Loads Dependent on The Material Thickness (mm)																	
	0.3	0.4	0.5	0.6	0.7	0.8	0.9	1.0	1.1	1.2	1.3	1.4	1.5	1.6	1.7	1.8	1.9	2.0
ESS [N]	11.1	17.5	25.2	34.6	45.6	58.6	73.5	90.6	109.9	131.7	156.0	183.0	212.9	245.7	281.6	320.7	363.3	409.3
EMX [N]	50.4	62.9	75.2	89.2	106.5	129.0	158.3	196.3	244.5	304.8	379.0	468.6	575.6	701.5	848.3	1017.5	1210.9	1430.4
LP [N]	48.0	101.2	171.6	258.3	360.6	477.6	608.5	752.4	908.5	1075.9	1253.9	1441.5	1638.0	1842.5	2054.1	2272.1	2495.6	2723.7

## Data Availability

The data are contained within the article.
